# Diminishing clinical impact for post-approval cancer clinical trials: A retrospective cohort study

**DOI:** 10.1371/journal.pone.0274115

**Published:** 2022-09-12

**Authors:** Charlotte Ouimet, Gauthier Bouche, Jonathan Kimmelman

**Affiliations:** 1 McGill University, Biomedical Ethics Unit, Montreal, QC, Canada; 2 The Anticancer Fund, Brussels, Belgium; University of Rennes 1, FRANCE

## Abstract

**Background:**

Once a drug gets FDA approved, researchers often attempt to discover new applications in different indications. The clinical impact of such post-approval activities is uncertain. We aimed to compare the clinical impact of research efforts started after approval with those started before for cancer drugs.

**Methods:**

We used Drugs@FDA to perform a retrospective cohort study of secondary approvals for cancer drugs that were initially FDA approved between 2005 and 2017. Clinicaltrials.gov was used to identify the beginning of each research trajectory that resulted in a secondary FDA approval. Each trajectory was classified as pre- or post-approval depending on if it was initiated before or after initial drug licensure. Clinical impact was assessed by comparing secondary approvals and NCCN off-label recommendations deriving from pre- vs. post-approval trajectories, pooled effect sizes, incidence, and level of evidence.

**Results:**

We identified 77 broad secondary approvals, 60 of which had at least 6 years follow-up. Of these, 9 (15%) resulted from post-approval trajectories, a proportion that is significantly lower than would be expected if the timing of research didn’t impact approval (McNemar’s test p = 0.001). Compared to pre-approval trajectories, approvals resulting from post-approval trajectories were for cancers with lower mean incidence (6.11 vs 14.83, p = 0.006) and were based on pivotal trials with smaller pooled effect sizes (0.69 vs 0.57, p = 0.02) that were less likely to be randomized (38.5% vs 64.1%, p = 0.145). We identified 69 NCCN off-label recommendations. The proportion stemming from post-approval trajectories was similar to that for pre-approval (56.5% vs. 43.5%). However, recommendations from post-approval trajectories were significantly more likely to involve rare diseases (76.7% vs 51.4%, p = 0.019) and nonsignificantly less likely to be based on level 1 evidence (11.6% vs 22.9%, p = 0.309).

**Conclusion:**

Secondary FDA approvals are less likely to result from post-approval trajectories and tend to be less impactful compared to approvals originating from research started before first FDA licensure. However, post-approval trajectories may be as likely to lead to NCCN recommendations for off-label use. Limitations of this work include our use of indirect measures of impact and limited follow-up time for trajectories. Our study protocol was pre-registered (https://osf.io/5g3jw/).

## Introduction

Drug development is notoriously slow and costly. In cancer, firms spend an estimated $648.0 million and 7.3 years to advance a new drug to regulatory approval [1]. Such cost and delay are partly driven by high rates of drug attrition. Given that many diseases share common pathophysiological origins, one way companies and academic investigators maximize the value of drug development is to test already approved drugs in different diseases (“drug repurposing”) [2]. Approximately 29% of all cancer clinical trials involve attempts to repurpose already approved cancer drugs for other cancers [3].

Drug repurposing is attractive in part because approved drugs have relatively well-established safety and dosing profiles, allowing for research at lower cost and with less innovation needed [4,5]. Post-approval trials also offer additional options for patients who may have exhausted established effective therapy. Even with these benefits, however, drug repurposing involves costs and potential burdens for research subjects. Prior studies of repurposing research efforts in cancer suggest successful research efforts are infrequent. For example, in one study, post-approval phase II and III trials were no more likely to meet their primary endpoint than traditional drug development trials [6]. In another study of cancer drugs approved by the FDA between 2005–2007, no indication-drug pairings for monotherapies launched into clinical trials after initial regulatory approval advanced to FDA approval within 5 years [7]; the same was true for combination therapies in this time frame [8]. Other studies reinforce these findings [9]. Some studies also suggest that post-approval clinical trials tend to use less rigorous research methodologies [10]. Owing to patent life, companies have incentives to pursue the most promising avenues of research early in a drug’s development. Despite additional market exclusivity being offered for secondary indications by the FDA, companies may lack strong incentive to invest in drug repurposing [11]. Drug companies may have difficulty enforcing market exclusivity for new indications [12,13]. Once the original patent expires and a generic becomes available, the generic version of the drug is often prescribed for all indications for which the brand name drug is approved regardless of market exclusivity [14]. In one analysis, 92.5% of extensions for new indications were authorized during the exclusivity period of the original product [15]. Together, these findings suggest that for all the economies of post-approval research, almost all the clinical value of new drugs is anticipated before drugs receive FDA approval and investing in drug repurposing may subject patients to the burdens of clinical research with limited potential for advancing medicine.

Clinical trials make use of limited research personnel and involve potential burdens for clinical trial participants. All else being equal, research systems should prioritize research that achieves the greatest clinical impact for this investment of resources and patient welfare. Evidence on the relative clinical impact of de novo vs. repurposing drug development can help inform the development of policies that promote an optimal balance between the two modes of drug development. It can also inform physician and/or decisions about where to invest their energies.

In what follows, we describe a retrospective cohort study comparing the proportion of cancer secondary approvals deriving from pre-approval vs. post-approval-initiated research efforts, as well as the characteristics of secondary approvals with respect to effect sizes, evidence quality and disease incidence. We hypothesized that secondary approvals originating from post-approval research would be less common, involve lower incidence cancers, and be based on weaker evidence than drug approvals deriving from pre-approval research efforts.

## Methods

### Objectives and definitions

Our primary objective was to compare the frequency of secondary approvals deriving from clinical research efforts launched before vs. after the first FDA approval of a drug. We define “secondary approvals” as cancer indications that were added to the FDA label after the first approval of a drug. A “trajectory” is defined as a series of one or more clinical trials testing a unique drug-indication pairing. “Pre-approval trajectories” are trajectories that were launched before the first FDA approval of the study drug. “Post-approval trajectories” are trajectories that were started after the first FDA approval of the study drug. To be clear, a trial might be launched after approval of a drug, but nevertheless belong to a trajectory that was launched pre-approval. A drug might also receive a secondary approval based on a trajectory that was launched pre-approval.

Secondary objectives included comparing indication incidence, effect sizes and quality of evidence for FDA approvals and evaluating NCCN guideline off-label recommendations originating from pre- vs. post-approval trajectories. Our study protocol was pre-registered on Open Science Framework (https://osf.io/5g3jw/).

### Drug sample

We created our sample of anti-cancer drug approvals by searching Drugs@FDA for all new molecular entities (NMEs) that received a primary anti-cancer drug approval between January 1^st^, 2005 and December 30^th^, 2017. We recorded all secondary approvals occurring after the drug’s first FDA approval. We excluded all adjuvant or neoadjuvant secondary approvals from the analysis. The 2017 cut-off date was chosen to capture at least three years of potential secondary approvals for all drugs in our sample. Drugs were excluded from further analysis if they i) did not receive any secondary approvals as of January 2021, or ii) treated symptoms of cancer or symptoms secondary to cancer treatments.

### Primary analysis: Timing of trajectory launch

All secondary approvals were classified based on whether their research trajectories were launched pre- vs. post-approval. Because many trajectories start in broad indications and narrow to sub-indications as they progress, we assigned each drug approval to an indication category using the broad indication categories used by the National Comprehensive Cancer Network (NCCN) guidelines. For example, since the guidelines for treating follicular lymphoma and mantle cell lymphoma are provided in NCCN’s guidelines for treating B-cell lymphoma, both these indications fall into the same indication category and are treated as one broad indication. Thus, a trial testing drug X in follicular lymphoma and a trial testing drug X in mantle lymphoma would be part of the same trajectory. This approach might lead to an undercount of approvals or recommendations but ensured the integrity of comparisons between pre- and post-approval trajectory impacts. For each secondary approval, we searched clinicaltrials.gov for the first trial (any phase) of its trajectory. For a trajectory to be categorized as “post-approval,” the first trial in the trajectory would have had to have been started after the initial FDA approval. For the remainder of the analyses, we studied the secondary approvals as written on the FDA labels instead of using broad indication categories.

### Disease incidence for indications receiving secondary approvals

We assessed the incidence of indications that ultimately received full approval based on the specific disease as written on the FDA label (e.g., in the above example, mantle cell lymphoma). The Orphan Drug Act assigns orphan status based on prevalence [16]. However, we were unable to find reliable prevalence data for all indications in our study. We therefore used disease incidence as a proxy for rarity. An indication is rare if it has an incidence less than 6 per 100 000 [17]. Cancer.net was used to find the projected number of cases for 2020 for each indication. When unavailable using this source, we obtained estimates from recent publications. Incidence was calculated using the population of the United States in 2020 (332 600 000) [18].

### Clinical value added

For each specific secondary approval and as a post hoc analysis, we searched France’s *Haute Authorité de Santé* database to identify the *amélioration du service médical rendu* (ASMR) score [19]. This score measures the severity of the disease treated, the efficacy of the drug, the undesirable effects of the drug and how the drug compares to what is already available. Approvals are given a score between 5 and 1, with 1 being that the approval presents major additional clinical value and 5 being that the approvals does not present any additional clinical value.

### Effect sizes and evidence quality for pivotal trials of secondary approvals

For each secondary approval, we identified the pivotal clinical trial from *Section 14*: *Clinical Studies* of the FDA labels of study drugs. The following design elements and results were extracted from clinicaltrials.gov registration records and/or publications for all pivotal trials to assess the quality of evidence: a) effect size for progression-free survival (PFS), b) whether the primary endpoint was a surrogate (e.g.: objective response rate) vs. clinical endpoint (e.g.: overall survival), c) sample size, d) use of randomization, and e) use of masking, f) sponsor. The latter four elements track design features widely associated with trial quality [20,21]. PFS is a weak surrogate in many situations in cancer and should not be assumed to necessarily reflect clinical impact [22]. However, it was selected for this analysis because it is the most frequently used endpoint in cancer trials and hence it maximized our ability to compare outcomes among strata. Trials were identified using NCT registration numbers where available, or by conducting searches on clinicaltrials.gov using the information on dosage, enrollment, trial location etc. When multiple trials were cited as pivotal for a single indication, the trial with the largest effect size for PFS was used for analysis. If effect size was not reported for any of the trials, the pivotal trial was picked based on discussion between two individuals (C.O., J.K.). We performed a meta-analysis of the effect size of PFS comparing pivotal trials for approvals originating from pre-approval vs post-approval trajectories. We appreciate that our meta-analyses involved a highly heterogenous sample of indications and drugs for secondary approvals. We nevertheless reasoned that our meta-analysis would offer a broad picture of the comparative magnitude of effect sizes observed in pre- vs. post-approval pivotal trials. Numerous other meta-research studies have been guided by a similar meta-analytic approach [23–26].

### Recommendations for off-label use

Another potential marker of clinical impact is recommendations for off-label use in clinical practice guidelines. Drug companies have weaker incentives to pursue secondary indication regulatory approvals late in the patent life of a new drug. Therefore, our primary endpoint (secondary approvals) might fail to capture the entirety of a repurposed drug’s clinical impact. We thus repeated the above analyses using off-label recommendations in the guidelines issued by the National Comprehensive Cancer Network (NCCN). NCCN guidelines are one of several compendia used to determine Medicare coverage for cancer treatments in the U.S. [27].

All NCCN guidelines downloaded on January 7^th^, 2020 were searched for recommendations involving off-label use of drugs in our sample. For each broad recommendation, we determined whether trajectories had been launched pre- vs. post-approval. We found disease incidence for each specific indication as above. We evaluated evidence quality by assessing whether the recommendations were supported by level 1 evidence, defined as a randomized control trial [28]. Because it is difficult to test rare cancers in randomized trials due to limitations in available patient population [29], we used a more permissive standard of evidence quality for rare cancers. We evaluated if recommendations for rare were supported by level 2 evidence (e.g. individual cohort study) as a more permissive standard of evidence quality [28].

### Analysis

For the primary endpoint, the secondary approvals from the subset of drugs in our sample approved between January 1^st^, 2005 and December 31^st^, 2014 were analyzed to allow for at least six years of follow-up for secondary approvals occurring in recent drugs. For secondary analyses, the approvals from all drugs approved in the timeframe of the study were included. We performed a descriptive analysis for all outcomes measured in this study. The above analyses were repeated using off-label recommendations in NCCN guidelines instead of FDA approvals.

We performed a sensitivity analysis to address differential follow-up time for pre- vs. post-approval trajectories (by definition, the former will afford greater follow-up time, which would bias against post-approval trajectories demonstrating impact through FDA approvals). We reperformed our primary endpoint analysis only using trajectories that are 6 years or shorter. Thus, if a secondary approval resulted from a trajectory launched eight years before the date of its approval, it was excluded from this analysis.

We used chi-square for independence and t-tests for inferential analyses of differences between proportions and between means, respectively. We performed a McNemar test to compare the drugs that received secondary approvals originating from pre-approval and/or post-approval trajectories. We also performed a meta-regression with categorical covariates comparing the PFS reported in pre- and post-approval pivotal trials. We defined p < 0.05 as significant and did not correct for multiple hypothesis testing due to the exploratory nature of inferential tests. All inferential tests that were performed are reported. All analysis was performed using RStudio version 1.3.1056 (RStudio, Inc).

### Ethics

This study did not entail interaction with or collecting data from research subjects. It is therefore exempt from IRB submission.

### Protocol modifications

Follow-up time was extended from January 2020 to January 2021 for secondary approvals. Instead of performing a risk of bias assessment, pivotal trial characteristics (randomization, blinding, clinical endpoint) were extracted to assess evidence quality supporting secondary approvals. Sponsorship of pivotal trials was also extracted. Due to available personnel, data collection was performed by CO with the consultation of JK and GB. Incidence was used as a proxy for prevalence since prevalence data weren’t widely available. We performed a post-hoc McNemar analysis to compare the drugs that received pre- and/or post- secondary approvals. We also performed a post-hoc analysis assessing the clinical value of each secondary approval.

## Results

Eighty-eight anti-cancer oncology drugs meeting eligibility for our study were approved by the FDA between January 1^st^, 2005 and December 31^st^, 2017. Thirty (34.1%) of these drugs had at least one secondary approval by January 1^st^, 2021, resulting in 84 secondary approvals that were classified into 77 broad indication categories (S1 Table).

Of the 58 drugs approved that had at least 6 years follow-up time, 22 drugs (37.9%) obtained at least one secondary approval, resulting in 67 specific secondary approvals that were classified into 60 broad indication categories.

**Fig 1:**
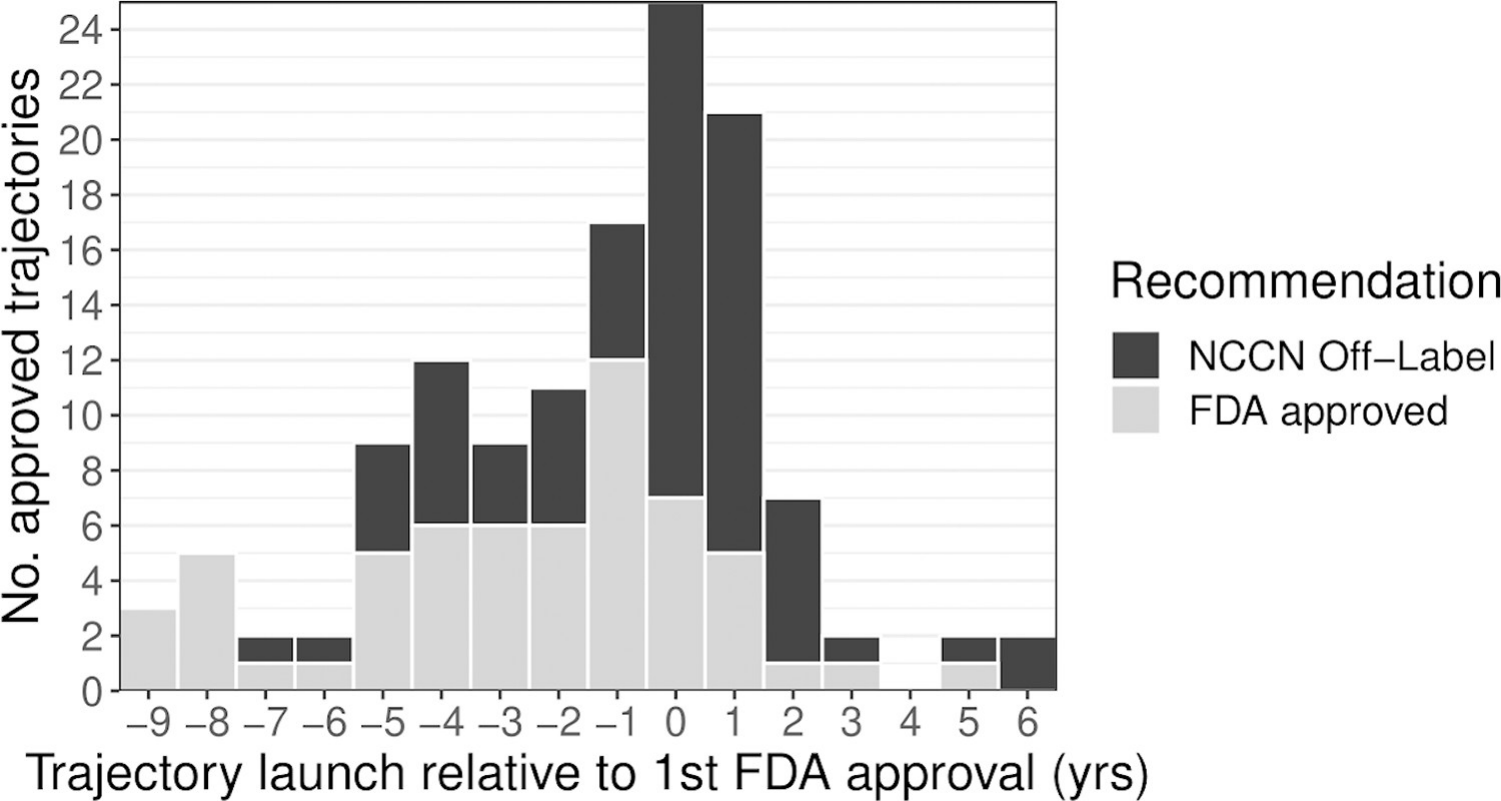
Trajectory launch date relative to FDA approval for secondary approvals and off-label recommendations. The date trajectory launch for each drug-indication pairing was graphed in comparison to the first FDA approval, which is represented by 0 on the x-axis. The light grey represents the beginning of trajectories that lead to secondary approvals while the dark grey represents the ones that lead to off-label recommendations in NCCN guidelines.

A lower proportion of drugs received a secondary approval from a post-approval trajectory than from a pre-approval trajectory. Twenty drugs (34.5%) had at least one secondary approval that originated from a pre-approval trajectory while 5 (8.6%) had at least one approval originating from a post-approval trajectory. Seventeen drugs (29.3%) only had secondary approvals originating from pre-approval trajectories while 2 drugs (3.5%) only had secondary approvals originating from post-approval trajectories. We performed a McNemar test, which indicated that the distribution of drugs with secondary approvals originating from pre- vs post-approval trajectories is more than would be expected by chance (p = 0.001). Similarly, a lower proportion of secondary approvals resulted from post-approval trajectories as compared with pre-approval (15% (n = 9) vs. 85% (n = 51)); (Fig 1). Post-approval trajectories led to 0.049 secondary approvals per year of available research time compared with 0.38 for pre-approval trajectories.

### Disease incidence, evidence quality, clinical value added and effect sizes for secondary approvals

In total, 38 out of 84 specific secondary approvals involved rare cancers. The median incidence of indications for secondary indications achieved from post- vs. pre-approval research were 2.78 and 7.34, respectively. Secondary approvals resulting from post-approval trajectories tended to involve rare cancer indications more frequently than secondary approvals originating from pre-approval trajectories; see Table 1.

**Table 1:** Characteristics of the cancers and pivotal trials for secondary approvals resulting from pre-approval compared to post-approval trajectories.

Characteristics	All new indications	New indications that resulted from pre-approval trajectories N = 69	New indications that resulted from post-approval trajectories N = 15	p-value
Cancer type				
Mean incidence	10.47	14.83	6.11	0.006
Proportion that are rare	38 (45.2%)	29 (47.5%)	9 (60%)	0.677
Pivotal Trials*				
Mean enrollment	434 (s = 381)	437 (s = 397)	364 (s = 300)	0.459
Randomized	46 (59.7%)	41 (64.1%)	5 (38.5%)	0.086
Double blinded	21 (27.3%)	19 (29.7%)	2 (15.4%)	0.475
Clinical endpoint	29 (37.7%)	25 (39.1%)	4 (30.8%)	0.804
Industry sponsor	74 (96.1%)	62 (96.9%)	12 (92.3%)	1

*Since some approvals were supported by the same clinical trial, our study included 64 pivotal trials for pre-approval indications and 13 trials for post-approval indications.

**Fig 2:**
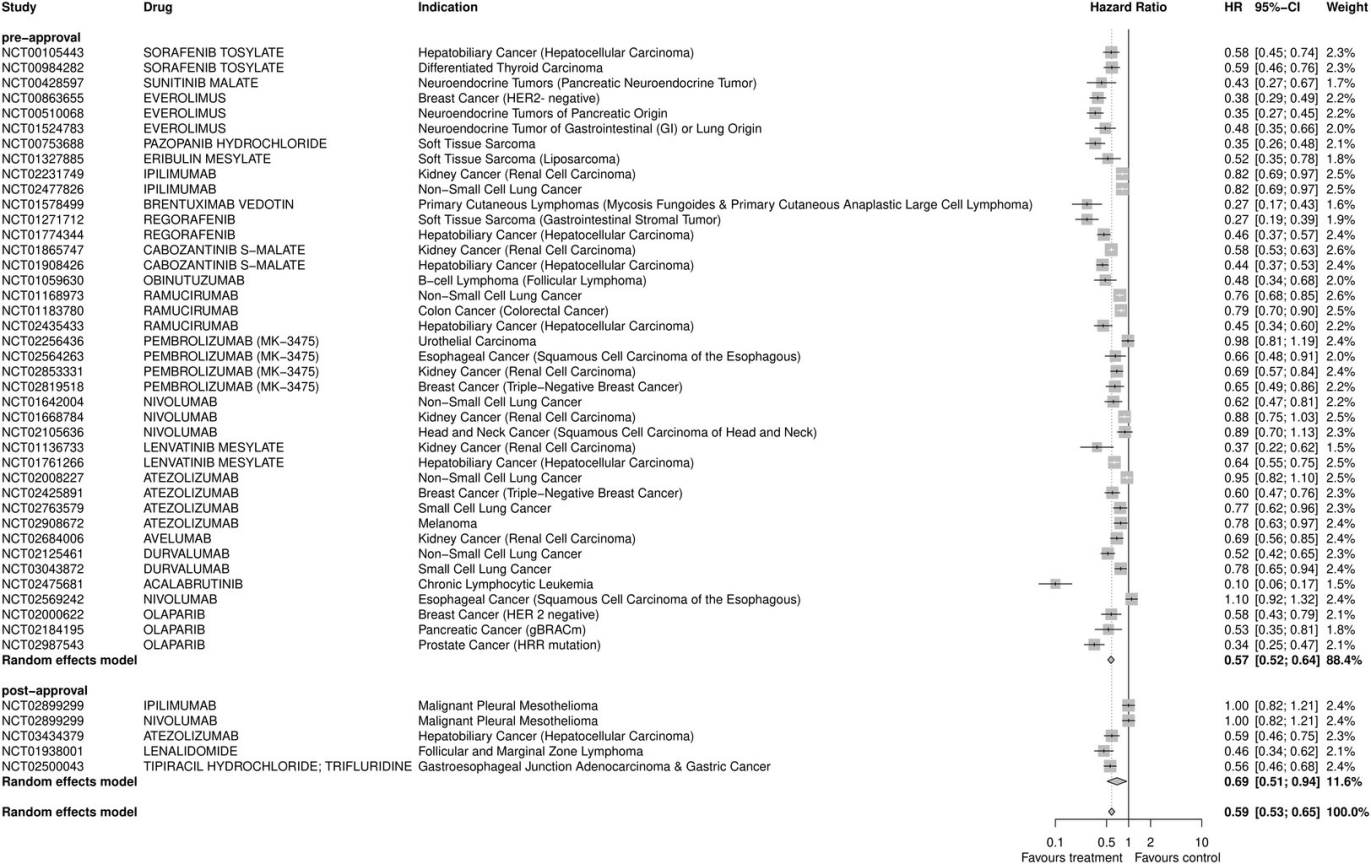
Meta-analysis of the effect size of pivotal trials. The forest plot shows the comparison of hazard ratios (HR) for progression free survival (PFS) for pivotal trials of FDA approvals originating from pre- vs post-approval trajectories (0.57 vs. 0.69, p = 0.02). Six pivotal trials for post-approval trajectories reported HR for PFS and were compared to thirty-six pivotal trials for pre-approval trajectories.

FDA approvals resulting from post-approval trajectories were less likely to be randomized, double-blinded or use a larger sample size but as likely to use a clinical endpoint as compared with pivotal trials for FDA approvals originating from pre-approval trajectories (Tables 1 and S2). Pivotal trials for approvals deriving from pre-approval trajectories were predominantly industry-funded as were those from post-approval trajectories. A meta-analysis of hazard ratio (HR) for progression-free survival showed smaller pooled effect sizes for pivotal trials resulting from post-approval efforts compared to pre-approval efforts with significant between group difference (Fig 2).

Fifty-seven clinical value assessments (ASMR scores) were available for the 84 secondary approvals captured (S1 Table). None of the FDA approvals were deemed to present major additional clinical value (Table 2).

**Table 2:** The clinical value added of secondary approvals resulting from pre-approval and post-approval trajectories.

	Clinical Value Added (ASMR scores)				
	Absent (5)	Minor (4)	Moderate (3)	Important (2)	Major (1)
Secondary approvals stemming from pre-approval trajectories	16	17	18	0	0
Secondary approvals stemming from post-approval trajectories	4	1	1	0	0

### Sensitivity analysis

We performed a sensitivity analysis using only the 30 broad secondary approvals for which development trajectories had been launched within six years of label revision. Secondary approvals were less likely to reflect post-approval trajectories as compared with pre-approval trajectories (23.3% vs. 76.7%).

### NCCN recommendations for off-label use

Sixty-nine off-label broad indication recommendations (78 specific recommendations) were found for the 21 drugs in our sample with at least 6 years follow-up time (S3 Table). Of trials cited to support NCCN recommendations for off-label use, 34% originating from pre-approval trajectories were industry funded vs. 24% for those originating from post-approval trajectories; further characteristics of cited trials are provided in the Appendix (S4 Table).

The proportion of broad recommendations originating from post-approval trajectories was similar to that from pre-approval trajectories (56.5% (n = 39) vs 43.5% (n = 30)) (Fig 1). Post-approval trajectories led to 0.21 NCCN off-label recommendations per year of available research time compared with 0.22 for pre-approval trajectories. No additional off-label recommendations were observed for drugs with less than 6 years follow-up.

Regarding incidence, 51 of 78 (65.4%) recommendations involved rare cancers. Off-label recommendations originating from post-approval research were significantly more likely to involve rare diseases than recommendations originating from pre-approval research; see Table 3. Overall, 16.7% of NCCN off-label recommendations were supported by level 1 evidence. More recommendations deriving from pre-approval trajectories were supported by level 1 evidence than from post-approval trajectories; see Table 3. The recommendations for rare cancers that came from post-approval trajectories were predominantly supported by at least level 2 evidence; see Table 3.

**Table 3:** Specific NCCN off-label recommendations.

	Originating from Pre-approval Trajectories N = 35	Originating from Post-approval Trajectories N = 43	p-value
Recommendations for rare indications	18 (51.4%)	33 (76.7%)	0.019
Level 1 evidence	8 (22.9%)	5 (11.6%)	0.309
Level 1 evidence (only non-rare indications)	5 (29.4%)	3 (30%)	1
Level 1 or 2 evidence (only rare indications)	16 (88.9%)	31 (93.9%)	0.923

## Discussion

Taken together, our findings suggest diminished returns for drug repurposing efforts within cancer that are started after a drug is already approved. First, most cancer indications added to a drug label originate from research begun before approval rather than after (85% vs 15%); these patterns hold when adjusting for differential follow-up time. Second, populations benefiting from secondary approvals tend to be smaller for drug repurposing efforts begun after drug approval. Third, evidence supporting additional approvals trended toward being of lower quality where development efforts are begun after approval compared to before (e.g., 64.1% of pivotal studies used randomization for pre-approval vs. 38.5% for post-approval research). Fourth, effect sizes tend to be smaller for approvals achieved from post-approval research. Last, our analysis of clinical practice guidelines accords with the above. Though the proportion of recommendations stemming from post-approval research was roughly equal to that for pre-approval, recommendations stemming from pre-approval trajectories were significantly less likely to involve rare cancers; we also observed a trend toward higher levels of evidence from pre-approval trajectories. Together, these findings are consistent with our hypothesis that most of a new drug’s eventual clinical impact is embodied in research started before drug approval.

Post-approval drug development may deliver value for patients with rare cancers. Over half of NCCN recommendations for off-label use stem from research efforts begun after regulatory approval, and over three quarters of these recommendations involve rare cancers. The finding that post-approval research results in off-label recommendations instead of new approvals is consistent with previous work showing limited conduct of phase III trials for research efforts launched after approval [30]. As described elsewhere [31], the clinical value of such recommendations is uncertain due to the generally low level of evidence supporting NCCN off-label recommendations.

Our findings are consistent with two mutually compatible dynamics. First, they might reflect that the prior probability of clinical impact diminishes with later trajectories. Second, they might reflect economic rewards of drug development. Incentives for drug development diminish as the patent life on a new drug dwindles. Drug companies also have strong incentives to prioritize drug development for indications with larger markets [2], and where evidence supporting clinical hypotheses is strongest. This would explain why secondary approvals from pre-approval research involve higher incident diseases and larger effect sizes. After a drug receives regulatory approval, the pressure to generate high quality evidence of efficacy diminishes, since recommendations in clinical practice guidelines are sufficient in many jurisdictions to qualify for reimbursement [27], though this varies among countries [32]. Yet companies still have some incentive, via the prospect of gaining 7 years of orphan exclusivity [11], to explore rare indications in the post-approval setting. The finding that evidence quality is diminished for approvals originating after approval also likely reflects that post-approval research is more likely to target rare cancers, where large, randomized trials face major challenges for recruitment [29].

Our study should be interpreted considering several limitations. First, our findings do not necessarily undermine the case for investing in repurposing research. As stated above, diminished impact for research efforts launched after FDA approval might reflect attenuated incentives for research [33], rather than the exhaustion of viable clinical hypotheses [11]. Possible approaches to encourage post-approval licensing include more expansive forms of market exclusivity (i.e. the “patent extension model”) [11]. Second, due to the inherently longitudinal nature of the question, pre-approval trajectories had more follow-up time than post-approval trajectories. Some post-approval trajectories launched may yet lead to new FDA approvals. We addressed this limitation using a sensitivity analysis and by normalizing our analysis by follow-up time. Even with consistent maturation time, pre-approval trajectories were more likely to lead to label revisions compared to post-approval trajectories. Third, this study used successful trajectories to retrospectively assess the impact of pre- vs post-approval research. Our analyses do not address the proportion of research efforts in pre- vs post-approval settings that lead to important advances in care. Fourth, our study is limited to cancer indications and did not consider non-cancer disease indications that might have advanced to an FDA approval. Similarly, our study did not capture instances of non-cancer drugs that received a secondary approval in a cancer indication (e.g., lanreotide, a drug from our cohort, was originally developed and approved for acromegaly before it was approved for a cancer indication). Last, our findings depend on our having dichotomized pre-approval and post-approval trajectories. Some pre-approval trajectories in our sample were more similar “post-approval” to post-approval trajectories in that drug companies may have been secure in the knowledge that their drug would soon be approved. Also, our findings understate the contrast between the productivity of pre- and post-approval research, since a drug’s first approval is not counted in our estimate of successful pre-approval trajectories.

In summary, secondary approvals resulting from clinical research efforts started after a drug is approved are uncommon. When they are achieved, they tend to be based on weaker evidence, produce smaller effect sizes, and involve rarer cancers than secondary approvals achieved from research initiated before approval. At the same time, recommendations for off-label treatment in clinical practice guidelines often derive from research initiated after drug approval. Our findings can help funders, researchers, patient advocate groups and patients themselves assess the potential impact of post-approval research efforts. As it stands, patient advocates should, all else being equal, advise their patients to participate in trials testing new drugs, rather than drugs that have already received approval, if the patients value participating in the testing of a treatment that will receive regulatory approval, involve a more prevalent cancer type, or be supported by good evidence. Our findings suggest that public policy and research oversight should either modify incentives to encourage more impactful post-approval research, or otherwise discourage post-approval research. Whatever advantages repurposing research has regarding prior clinical knowledge do not appear to translate to higher research efficiencies.

## Supporting information

S1 Table Secondary FDA Approvals resulting from pre- or post- approval research efforts. (PDF)

S2 Table Pivotal trials supporting specific secondary FDA approvals. (PDF)

S3 Table Off-label recommendations in NCCN Guidelines resulting from pre- or post-approval trajectories. (PDF)

S4 Table Evidence cited to support off-label recommendations in NCCN guidelines. (PDF)
