# Supplementary material for: Diminishing clinical impact for post-approval cancer clinical trials: A retrospective cohort study
Source: PLoS One. 2022 Sep 12;17(9):e0274115. doi: 10.1371/journal.pone.0274115 (PMC9467301; doi:10.1371/journal.pone.0274115)
Supplement: S1 Table — (PDF) [file pone.0274115.s001.pdf]

S1 Table: Secondary FDA Approvals Resulting from Pre- or Post- Approval Research Efforts

Initial Approval	Drug	Indication (not grouped)	Indications (Grouped)	Pre (0), Post (1)	First Clinical Trial in Trajectory	First Clinical Trial Start Date	Clinical Value Added*
2005-12-01	Sorafenib tosylate	Hepatocellular carcinoma	Hepatobiliary Cancer	0	NCT00044512	2002-08-01	4
		Thyroid carcinoma - differentiated	Thyroid Cancer	0	NCT00095693	2004-10-01	4
2005-12-27	Lenalidomide	Mantle cell lymphoma	B-Cell Lymphoma	1	NCT00419250	2006-12-01	5
		Follicular lymphoma					5
		Marginal zone lymphoma					-
2006-01-26	Sunitinib malate	Pancreatic neuroendocrine tumor - well differentiated	Neuroendocrine Tumors	0	NCT00056693	2003-04-01	5
2008-03-20	Bendamustine hydrochloride	B-cell non-Hodgkin lymphoma	B-Cell Lymphoma	0	NCT01456351	2003-09-01	5
2009-03-30	Everolimus	Breast Cancer - HER2 negative	Breast Cancer	0	NCT00255788	2005-01-19	5
		Neuroendocrine tumor of gastrointestinal origin	Neuroendocrine Tumors	0	NCT00655655	2004-12-01	5
		Neuroendocrine tumor of pancreatic origin					4
		Neuroendocrine tumor of lung origin					-
2009-10-19	Pazopanib hydrochloride	Soft tissue sarcoma - not adipocytic	Soft Tissue Sarcoma	0	NCT00297258	2005-11-01	5
2009-11-05	Romidepsin	Peripheral T-cell lymphoma	T-cell Lymphoma	0	NCT00007345	2001-03-08	N/A
2010-11-15	Eribulin mesylate	Liposarcoma	Soft Tissue Sarcoma	0	NCT00413192	2007-01-01	3
2011-03-25	Ipilimumab	Renal cell carcinoma	Kidney Cancer	0	NCT00057889	2003-02-01	3
		Colorectal cancer - microsatellite instability high, or mismatch repair deficient	Colon Cancer	0	NCT00047164	2002-09-01	-
		Hepatocellular carcinoma	Hepatobiliary Cancer	1	NCT01658878	2012-09-26	-
		Malignant pleural mesothelioma	Malignant Pleural Mesothelioma	1	NCT03048474	2016-09-01	-
		Non-small cell lung cancer	Non-Small Cell Lung Cancer	0	NCT00039091	2002-03-01	4
2011-08-19	Brentuximuab vedotin	Mycosis fungoides - Cd30 expressing	Primary Cutaneous Lymphomas	0	NCT01396070	2011-05-01	4
		Primary cutaneous anaplastic large cell lymphoma					3
2011-08-17	Vemurafenib	Erdheim chester disease (with BRAF V600 mutation)	Erdheim Chester Disease	1	NCT02089724	2014-03-01	-
2012-09-27	Regorafenib	Gastrointestinal stromal tumor	Soft Tissue Sarcoma	0	NCT01068769	2010-02-01	4
		Hepatocellular carcinoma	Hepatobiliary Cancer	0	NCT01003015	2009-09-01	4

2012-11-29	Cabozantinib s-malate	Renal cell carcinoma	Kidney Cancer	0	NCT01100619	2010-04-01	3
		Hepatocellular carcinoma	Hepatobiliary Cancer	0	NCT01658878	2012-09-26	4
2012-02-08	Pomalidomide	AIDS related Kaposi Sarcoma	AIDS Related Kaposi Sarcoma	0	NCT01495598	2012-01-10	-
2013-05-29	Dabrafenib mesylate	Non-small cell lung cancer BRAF V600E mutation	Non-Small Cell Lung Cancer	0	NCT01336634	2011-06-20	5
		Anaplastic thyroid cancer with BRAF V600E mutation	Thyroid Cancer	0	NCT01534897	2012-07-01	-
2013-05-29	Trametinib dimethyl sulfoxide	Non-small cell lung cancer BRAF V600E mutation	Non-Small Cell Lung Cancer	0	NCT01362296	2011-09-01	5
		Anaplastic thyroid cancer with BRAF V600E mutation	Thyroid Cancer	0	NCT01438554	2011-10-01	-
2013-11-01	Obinutuzumab	Follicular lymphoma	B-Cell Lymphoma	0	NCT01287741	2011-07-26	5
2013-11-13	Ibrutinib	Chronic lymphocytic leukemia	Chronic Lymphocytic Leukemia	0	NCT01105247	2010-05-01	3
		Waldenstrom macroglobulinemia	Waldenstrom Macroglobulinemia	0	NCT01109069	2010-06-01	-
2014-04-21	Ramucirumab	Non-small cell lung cancer	Non-Small Cell Lung Cancer	0	NCT00735696	2009-01-01	5
		Colorectal Cancer	Colon Cancer	0	NCT00862784	2009-04-01	5
		Hepatocellular carcinoma	Hepatobiliary Cancer	0	NCT00627042	2008-02-01	-
2014-09-04	Pembrolizumab	Non-small cell lung cancer	Non-Small Cell Lung Cancer	0	NCT01840579	2013-04-26	4
		Small Cell Lung Cancer	Small Cell Lung Cancer	0	NCT01840579	2013-04-26	-
		Head and Heck Squamous Cell Cancer	Head and Neck Cancer	0	NCT01986426	2013-11-01	3
		Hodgkin lymphoma	Hodgkin Lymphoma	0	NCT01953692	2013-11-22	4
		Primary Mediastinal Large b-cell lymphoma	B-Cell Lymphoma	0	NCT01953692	2013-11-22	-
		Urothelial Carcinoma	Bladder Cancer	0	NCT02043665	2013-12-18	4
		Bacillus Calmette-Guerin (BCG)-unresponsive, high-risk, non-muscle invasive bladder cancer (NMIBC)					-
		Colorectal Cancer - microsatellite instability high or mismatch repairer deficient	Colon Cancer	0	NCT01174121	2010-08-26	4
		Gastric Cancer - PD-L1 expressing	Gastric Cancer	0	NCT02013154	2014-01-01	-
		Squamous cell carcinoma of the esophagus - PD-L1 expressing	Esophageal Cancer	0	NCT02013154	2014-01-01	3
		Gastro-esophageal junction adenocarcinoma - PD-L1 expressing					3
		Cervical - PD-L1 expressing	Cervical Cancer	1	NCT02628067	2015-12-18	-

		Hepatocellular carcinoma	Hepatobiliary Cancer	0	NCT02178722	2014-07-17	-
		Merkel Cell Carcinoma	Merkel Cell Carcinoma	1	NCT02267603	2014-11-25	-
		Renal Cell Carcinoma	Kidney Cancer	0	NCT02009449	2013-11-15	3
		Endometrial Carcinoma - not MSI-H of dMMR	Uterine Neoplasm	0	NCT02178722	2014-07-17	3
		Triple-Negative Breast Cancer - PD-L1 expressing	Breast Cancer	0	NCT01042379	2010-03-01	3
		Metastatic cutaneous squamous cell carcinoma	Cutaneous Melanoma	1	NCT02376699	2015-02-28	-
2014-12-19	Olaparib	Breast Cancer HER2 neg, deleterious gBRACm	Breast Cancer	0	NCT00494234	2007-06-15	5
		Pancreatic adenocarcinoma, gBRACm mutated	Pancreatic Cancer	0	NCT00515866	2007-08-01	5
		Prostate Cancer, HRR gene mutation, castration resistant prostate cancer	Prostate Cancer	0	NCT01078662	2010-02-21	4
2014-12-22	Nivolumab	Non-small cell lung cancer	Non-Small Cell Lung Cancer	0	NCT00441337	2006-08-01	4
		Renal cell carcinoma	Kidney Cancer	0	NCT00441337	2006-08-01	3
		Hodgkin lymphoma	Hodgkin Lymphoma	0	NCT01592370	2012-06-27	5
		Head and Heck Squamous Cell Cancer	Head and Neck Cancer	0	NCT02105636	2014-05-01	-
		Urothelial Carcinoma	Bladder Cancer	1	NCT02496208	2015-07-09	-
		Colorectal Cancer - microsatellite instability high or mismatch repairer deficient	Colon Cancer	0	NCT00441337	2006-08-01	5
		Hepatocellular carcinoma	Hepatobiliary Cancer	0	NCT01658878	2012-09-26	-
		Squamous cell carcinoma of the esophagus	Esophageal Cancer	0	NCT02267343	2014-10-01	3
		Malignant Pleural Mesothelioma	Malignant Pleural Mesothelioma	1	NCT01822509	2015-07-01	4
2015-02-13	Lenvatinib mesylate	Renal cell carcinoma	Kidney Cancer	0	NCT01136733	2010-08-05	3
		Hepatocellular carcinoma	Hepatobiliary Cancer	0	NCT00946153	2009-07-24	-
		Endometrial Carcinoma - that is not microsatellite instability-high (MSI-H) or mismatch repair deficient (dMMR)	Uterine Neoplasm	0	NCT01111461	2010-03-01	3
2015-09-22	Tipiracil hydrochloride; Trifluridine	Gastro-esophageal junction adenocarcinoma	Esophageal Cancer	1	NCT03686488	2018-11-29	5
		Gastric Cancer	Gastric Cancer	1	NCT02500043	2015-12-01	5
2016-04-11	Venetoclax	Acute Myeloid leukemia	Acute Myeloid Leukemia	0	NCT01211457	2010-06-17	3

2016-05-18	Atezolizumab	Non-small cell lung cancer	Non-Small Cell Lung Cancer	0	NCT01846416	2013-05-30	4
		Triple-Negative Breast Cancer - PD-L1 expressing	Breast Cancer	0	NCT01898117	2013-07-01	5
		Small Cell Lung Cancer	Small Cell Lung Cancer	0	NCT02748889	2016-03-01	4
		Hepatocellular carcinoma	Hepatobiliary Cancer	1	NCT03170960	2017-09-05	3
		Melanoma- BRAF V600 +	Melanoma	0	NCT01455103	2011-11-01	-
2016-12-19	Rucaparib	Prostate Cancer - BRCA mutation	Prostate Cancer	1	NCT02952534	2017-02-15	-
2017-03-23	Avelumab	Urothelial Carcinoma	Bladder Cancer	0	NCT02603432	2016-04-25	3
		renal cell carcinoma	Kidney Cancer	0	NCT02493751	2015-10-15	5
2017-05-01	Durvalumab	Non-small cell lung cancer	Non-Small Cell Lung Cancer	0	NCT02000947	2013-10-25	-
		Small Cell Lung Cancer	Small Cell Lung Cancer	0	NCT02701400	2016-04-14	4
2017-10-31	Acalabrutinib	Chronic Lymphocytic leukemia	Chronic Lymphocytic Leukemia	0	NCT02029443	2014-01-01	3

* Level of clinical value. 5 is absent, 4 is minor, 3 is moderate, 2 is important and 1 is major
