# Supplementary material for: Diminishing clinical impact for post-approval cancer clinical trials: A retrospective cohort study
Source: PLoS One. 2022 Sep 12;17(9):e0274115. doi: 10.1371/journal.pone.0274115 (PMC9467301; doi:10.1371/journal.pone.0274115)
Supplement: S2 Table — (PDF) [file pone.0274115.s002.pdf]

S2 Table: Pivotal Trials Supporting Specific Secondary FDA Approvals

Drug	Indication (not grouped)	Pivotal Trial	Sponsor (industry 1, other 0)	Phase	Randomization	Double Blinded	Clinical Endpoint	Enrollment
Sorafenib tosylate	Hepatocellular carcinoma	NCT00105443	1	III	1	1	1	602
	Thyroid carcinoma - differentiated	NCT00984282	1	III	1	1	0	417
Lenalidomide	Mantle cell lymphoma	NCT00737529	1	II	0	0	0	134
	Follicular lymphoma	NCT01938001	1	III	1	1	0	358
	Marginal zone lymphoma	*NCT01938001						
Sunitinib malate	Pancreatic neuroendocrine tumor - well differentiated	NCT00428597	1	III	1	1	0	171
Bendamustine hydrochloride	B-cell non Hodgkin lymphoma	NCT00139841	1	III	0	0	0	103
Everolimus	Breast Cancer - HER2 negative	NCT00863655	1	III	1	1	0	724
	Neuroendocrine tumor of gastrointestinal origin	NCT01524783	1	III	1	1	0	302
	Neuroendocrine tumor of pancreatic origin	NCT00510068	1	III	1	1	0	410
	Neuroendocrine tumor of lung origin	*NCT01524783						
Pazopanib hydrochloride	Soft tissue sarcoma - not adipocytic	NCT00753688	1	III	1	1	0	369
Romidepsin	Peripheral T-cell lymphoma	NCT00426764	1	II	0	0	0	131
Eribulin mesylate	Liposarcoma	NCT01327885	1	III	1	0	1	446
Ipilimumab	Renal cell carcinoma	NCT02231749	1	III	1	0	1	1390
	Colorectal cancer - microsatellite instability high, or mismatch repair deficient	NCT02060188	1	II	0	0	0	340
	Hepatocellular carcinoma	NCT01658878	1	I/II	0	0	0	154
	Malignant pleural mesothelioma	NCT02899299	1	III	1	0	1	605
	Non-small cell lung cancer	NCT02477826	1	III	1	0	1	2220
Brentuximuab vedotin	Mycosis fungoides - Cd30 expressing	NCT01578499	1	III	1	0	0	131
	Primary cutaneous anaplastic large cell lymphoma	*NCT01578499						
Vemurafenib	Erdheim chester disease (with BRAF V600 mutation)	NCT01524978	1	II	0	0	0	22
Regorafenib	Gastrointestinal stromal tumor	NCT01271712	1	III	1	1	0	199
	Hepatocellular carcinoma	NCT01774344	1	III	1	1	1	573
Cabozantinib s-malate	Renal cell carcinoma	NCT01865747	1	III	1	0	0	375
	Hepatocellular carcinoma	NCT01908426	1	III	1	1	1	707
Pomalidomide	AIDS related Kaposi Sarcoma	NCT01495598	0	I/II	0	0	0	28
Dabrafenib mesylate	Non-small cell lung cancer BRAF V600E mutation	NCT01336634	1	II	0	0	0	174

	Anaplastic thyroid cancer with BRAF V600E mutation	NCT02034110	1	II	0	0	0	23
Trametinib dimethyl sulfoxide	Non-small cell lung cancer BRAF V600E mutation	*NCT01336634						
	Anaplastic thyroid cancer with BRAF V600E mutation	*NCT02034110						
Obinutuzumab	Follicular lymphoma	NCT01059630	1	III	1	0	0	321
Ibrutinib	Chronic lymphocytic leukemia	NCT01105247	1	I/II	0	0	0	48
	Waldenstrom macroglobulinemia	NCT01614821	0	II	0	0	0	63
Ramucirumab	Non-small cell lung cancer	NCT01168973	1	III	1	1	1	1253
	Colorectal Cancer	NCT01183780	1	III	1	1	1	1072
	Hepatocellular carcinoma	NCT02435433	1	III	1	1	1	292
Pembrolizumab	Non-small cell lung cancer	NCT01295827	1	I	1	0	0	280
	Small Cell Lung Cancer	NCT02628067	1	II	0	0	0	59
	Head and Heck Squamous Cell Cancer	NCT01848834	1	I	0	0	0	174
	Hodgkin lymphoma	NCT02453594	1	II	0	0	0	211
	Primary Mediastinal Large b-cell lymphoma	NCT02576990	1	II	0	0	0	53
	Urothelial Carcinoma	NCT02256436	1	III	1	0	1	542
	Bacillus Calmette-Guerin (BCG)-unresponsive, high-risk, non-muscle invasive bladder cancer (NMIBC)	NCT02625961	1	II	0	0	0	96
	Colorectal Cancer - microsatellite instability high or mismatch repairer deficient	NCT02460198	1	II	0	0	0	124
	Gastric Cancer -PD-L1 expressing	NCT02335411	1	II	0	0	0	315
	Squamous cell carcinoma of the esophagus - PD-L1 expressing	NCT02564263	1	III	1	0	1	628
	Gastro-esophageal junction adenocarcinoma - PD-L1 expressing	*NCT02564263						
	Cervical - PD-L1 expressing	NCT02628067	1	II	0	0	0	98
	Hepatocellular carcinoma	NCT02702414	1	II	0	0	0	150
	Merkel Cell Carcinoma	NCT02267603	0	II	0	0	0	50
	Renal Cell Carcinoma	NCT02853331	1	III	1	0	1	861
	Endometrial Carcinoma - not MSI-H of dMMR	NCT02501096	1	I/II	0	0	0	108
	Triple-Negative Breast Cancer - PD-L1 expressing	NCT02819518	1	III	1	1	1	882
	Metastatic cutaneous squamous cell carcinoma	NCT03284424	1	II	0	0	0	150

Olaparib	Breast Cancer HER2 neg, deleterious gBRACm	NCT02000622	1	III	1	0	0	302
	Pancreatic adenocarcinoma, gBRACm mutated	NCT02184195	1	III	1	1	0	154
	Prostate Cancer, HRR gene mutation, castration resistant prostate cancer	NCT02987543	1	III	1	0	0	387
Nivolumab	Non-small cell lung cancer	NCT01642004	1	III	1	0	1	325
	Renal cell carcinoma	NCT01668784	1	III	1	0	1	821
	Hodgkin lymphoma	NCT02181738	1	II	0	0	0	294
	Head and Heck Squamous Cell Cancer	NCT02105636	1	III	1	0	1	361
	Urothelial Carcinoma	NCT02387996	1	II	0	0	0	270
	Colorectal Cancer - microsatellite instability high or mismatch repairer deficient	NCT02060188	1	II	0	0	0	74
	Hepatocellular carcinoma	NCT01658878	1	I/II	0	0	0	1097
	Squamous cell carcinoma of the esophagus	NCT02569242	1	III	1	0	1	390
	Malignant Pleural Mesothelioma	NCT02899299	1	III	1	0	1	605
Lenvatinib mesylate	Renal cell carcinoma	NCT01136733	1	I/II	1	0	0	153
	Hepatocellular carcinoma	NCT01761266	1	III	1	0	1	954
	Endometrial Carcinoma - that is not microsatellite instability-high (MSI-H) or mismatch repair deficient (dMMR)	NCT02501096	1	I/II	0	0	0	108
Tipiracil hydrochloride; Trifluridine	Gastro-esophageal junction adenocarcinoma	NCT02500043	1	III	1	1	1	506
	Gastric Cancer	*NCT02500043						
Venetoclax	Acute Myeloid leukemia	NCT02203773	1	I	0	0	1	115
Atezolizumab	Non-small cell lung cancer	NCT02008227	1	III	1	0	1	850
	Triple-Negative Breast Cancer - PD-L1 expressing	NCT02425891	1	III	1	1	1	900
	Small Cell Lung Cancer	NCT02763579	1	III	1	1	1	403
	Hepatocellular carcinoma	NCT03434379	1	III	1	0	1	480
	Melanoma- BRAF V600 +	NCT02908672	1	III	1	1	0	514
Rucaparib	Prostate Cancer - BRCA mutation	NCT02952534	1	II	0	0	0	360
Avelumab	Urothelial Carcinoma	NCT01772004	1	I	0	0	0	242
	Renal cell carcinoma	NCT02684006	1	III	1	0	1	886
Durvalumab	Non-small cell lung cancer	NCT02125461	1	III	1	1	1	713
	Small Cell Lung Cancer	NCT03043872	1	III	1	0	1	988
Acalabrutinib	Chronic Lymphocytic leukemia	NCT02475681	1	III	1	0	0	535

*Pivotal trials that were cited more than once
