# Supplementary material for: Diminishing clinical impact for post-approval cancer clinical trials: A retrospective cohort study
Source: PLoS One. 2022 Sep 12;17(9):e0274115. doi: 10.1371/journal.pone.0274115 (PMC9467301; doi:10.1371/journal.pone.0274115)
Supplement: S3 Table — (PDF) [file pone.0274115.s003.pdf]

S3 Table: Off-label Recommendations in NCCN Guidelines resulting from Pre- or Post- Approval Trajectories

Initial Approval	Drug	Indication (Specific)	Indication (Grouped)	Pre approval (0), Post approval (1)	Primary Trial	(Primary) Start clinical trial
2005-12-01	Sorafenib tosylate	Acute Myeloid Leukemia	Acute Myeloid Leukemia	0	NCT02867891	2001-03-01
		Gastrointestinal Stromal Tumor	Soft Tissue Sarcoma	0	NCT00245102	2005-09-01
		Desmoid Tumor				
		Angiosarcoma				
		Solitary Fibrous tumor				
		Chordoma	Bone Cancer	1	NCT00330421	2006-06-01
		Osteosarcoma				
2005-12-27	Lenalidomide	CLL/SLL	Chronic Lymphocytic Leukemia/Small lymphocytic lymphoma	0	NCT00267059	2005-12-01
		Systemic Light Chain Amyloidosis	Systemic Light Chain Amyloidosis	0	NCT00166413:	2005-04-01
		Primary CNS lymphoma	Central Nervous System Cancer	0	NCT00036894	2002-03-01
		Peripheral T cell lymphomas - Adult T cell leukemia/lymphoma (first - larger)	T cell lymphoma	1	NCT00322985	2006-06-01
		Hodgkin Lymphoma	Hodgkin lymphoma	1	NCT00478959	2006-12-01
2006-01-26	Sunitinib malate	Meningiomas	Central Nervous System Cancer	1	NCT00387920	2006-10-01
		Thyroid carcinoma	Thyroid carcinoma	1	NCT00381641	2006-08-08
		Thymic Carcinoma	Thymomas and Thymic Carcinomas	1	NCT01306045	2011-02-08
		Chordoma	Bone cancer	1	NCT00474994	2007-04-01
2008-03-20	Bendamustine hydrochloride	SCLC	Small Cell Lung Cancer	0	NCT00168922:	2001-02-01
		Multiple Myeloma	Multiple Myeloma	1	NCT00889798	2009-04-01
		Hodgkin Lymphoma	Hodgkin Lymphoma	1	NCT00705250	2008-06-01
		Peripheral T -cell Lymphoma	T-cell lymphomas	1	NCT00880815	2009-02-17
		ALCL		1		
		WM/LLT	Waldenstrom Macroglobulinemia and Lymphoplasmacytic Lymphoma	1	NCT01110135	2010-08-01
2009-03-30	Everolimus	Glioma/Pilocytic	Central Nervous System Cancer	0	NCT00107237	2003-10-01
		Meningiomas		0		
		Thyroid carcinoma	Thyroid carcinoma	0	NCT00655655	2004-12-01
		Endometrial Carcinoma	Uterine Neoplasms	0	NCT00087685	2004-06-01
		Gastrointestinal Stromal Tumor	Soft Tissue Sarcoma	0	NCT01275222	2002-11-01
		Perivascular Epithelioid Cell Tumor				
		Thymoma	Thymomas and Thymic Carcinomas	1	NCT02049047	2011-02-01
		Thymic Carcinoma		1		
		Osteosarcoma	Bone Cancer	1	NCT01830153	2010-04-01
		Hodgkin Lymphoma	Hodgkin Lymphoma	1	NCT00918333	2009-06-01

		WM/LLT	Waldenstrom Macroglobulinemia and Lymphoplasmacytic Lymphoma	1	NCT00918333	2009-06-01
2009-10-19	Pazopanib hydrochloride	Epithelial ovarian/Fallopian tube/Peritoneal cancer	Ovarian Cancer	0	NCT00281632	2006-03-01
		Thyroid carcinoma	Thyroid carcinoma	0	NCT00625846	2008-02-22
		Uterine Sarcoma	Uterine Neoplasms	0	NCT00430781	2006-11-01
2010-11-15	Eribulin mesylate	Uterine sarcoma	Uterine Neoplasms	0	NCT00410553	2006-11-14
2011-03-25	Ipilimumab	SCLC	Small Cell Lung Cancer	0	NCT00527735	2008-02-01
		Melanoma Brain Metastases	Central Nervous System Cancer	1	NCT01950195	2013-09-01
		Uveal melanoma	Uveal melanoma	1	NCT01585194	2012-11-29
2011-08-17	Vemurafenib	BRAF V600+ NSCLC	Non-Small Cell Lung Cancer	1	NCT02314481	2017-05-12
		Brain Metastases	Central Nervous System Cancer	1	NCT01748149	2014-04-29
		Thyroid carcinoma	Thyroid carcinoma	1	NCT01709292	2012-11-07
		Hairy Cell Leukemia	Hairy Cell Leukemia	1	NCT01711632	2012-10-01
2011-08-19	Brentuximab vedotin	Diffuse Large B-cell lymphoma	B-cell lymphomas	0	NCT01421667	2011-08-01
2012-02-08	Pomalidomide	Primary CNS lymphoma	Central Nervous System Cancer	0	NCT01421524	2011-09-12
		Systemic light chain amyloidosis	Systemic light chain amyloidosis	0	NCT01510613	2012-02-01
2012-09-27	Regorafenib	Osteosarcoma	Bone cancer	1	NCT02048371	2014-07-01
2012-11-29	Cabozantinib s-malate	NSCLC	Non-Small Cell Lung Cancer	0	NCT00596648	2007-12-01
2013-05-29	Trametinib dimethyl sulfoxide + Dabrafenib mesylate	Rectal Cancer	Rectal Cancer	0	NCT01740648	2012-11-26
2013-05-29	Trametinib dimethyl sulfoxide	Uveal melanoma	Uveal melanoma	0	NCT01328106	2010-11-01
		Ovarian Cancer	Ovarian Cancer	1	NCT01902173	2013-07-19
		Colon Cancer	Colon cancer	1	NCT01902173	2013-07-19
2013-11-13	Ibrutinib	Hairy Cell Leukemia	Hairy Cell Leukemia	0	NCT01841723	2013-04-30
		Primary CNS lymphoma	Central Nervous System Cancer	1	NCT02203526	2014-08-14
2014-09-04	Pembrolizumab	Brain Metastases	Central Nervous System Cancer	0	NCT01174121	2010-08-26
		Prostate Cancer	Prostate Cancer	0	NCT02009449	2013-11-15
		Pancreatic Cancer	Pancreatic Adenocarcinoma	0	NCT01174121	2010-08-26
		Occult Primary	Occult Primary	1	NCT02538510	2015-10-08
		Anal Cancer	Anal Carcinoma	1	NCT02628067	2015-12-18
		CLL/SLL	Chronic Lymphocytic Leukemia/Small lymphocytic lymphoma	1	NCT02362035	2015-02-01
		High-Risk Gestational Trophoblastic Neoplasia	Gestational Trophoblastic Neoplasia	1	NCT04303884	2020-06-01
		Malignant Pleural Mesothelioma	Malignant Pleural Mesothelioma	1	NCT02399371	2015-03-31
		Alveolar Soft Part Sarcoma	Soft Tissue Sarcoma	1	NCT02301039	2015-03-01
		Undifferentiated Pleomorphic Sarcoma				

		Thymic Carcinoma	Thymomas and Thymic Carcinomas	1	NCT02364076	2015-03-01
		Bone cancer with mismatch repair	Bone cancer	1	NCT02301039	2015-03-01
		Mycosis Fungosis	Primary Cutaneous Lymphomas	1	NCT02243579	2014-10-15
		Extranodal NK	T-cell lymphomas	1	NCT02362997	2015-04-01
		Testicular Cancer	Testicular cancer	1	NCT02499952	2016-01-01
		Uveal melanoma	Uveal melanoma	1	NCT02359851	2015-05-01
		Penile Cancer	Penile cancer	1	NCT02721732	2016-08-15
2014-12-22	Nivolumab	Brain Metastases (melanoma, NSCLC Origin),	Central Nervous System Cancer	0	NCT02460068	2012-12-01
		Richter Transformation	Chronic Lymphocytic Leukemia/Small lymphocytic lymphoma	0	NCT01822509	2013-05-16
		Merkel Cell Carcinoma	Merkel Cell Carcinoma	0	NCT02196961	2014-06-01
		Extranodal NK	T-cell lymphomas	0	NCT01716806	2012-10-31
		Uveal melanoma	Uveal melanoma	0	NCT01585194	2012-11-29
		Anal Cancer	Anal Carcinoma	1	NCT02314169	2015-05-01
