# Supplementary material for: Diminishing clinical impact for post-approval cancer clinical trials: A retrospective cohort study
Source: PLoS One. 2022 Sep 12;17(9):e0274115. doi: 10.1371/journal.pone.0274115 (PMC9467301; doi:10.1371/journal.pone.0274115)
Supplement: S4 Table — (PDF) [file pone.0274115.s004.pdf]

S4 Table: Evidence Cited to Support Off-label Recommendations in NCCN guidelines

Drug	Indication (Specific)	PMID	Sponsor (Industry or not)	Phase	Randomized	Double blinded	Enrollment	Clinical Endpoint
Sorafenib tosylate	Acute Myeloid Leukemia	PMID: 23613521	0	I/II	0	0	60	0
	Gastrointestinal Stromal Tumor	PMID: 22270258	0	II	0	0	39	0
	Desmoid Tumor	PMID: 21447727	0	N/A	N/A	N/A	26	
	Angiosarcoma	PMID: 19451436	0	II	0	0	147	0
	Solitary Fibrous tumor	PMID: 24005614	0	II	0	0	96	0
	Chordoma	PMID: 26202596	0	II	0	0	96	0
	Osteosarcoma	PMID: 25498219	0	II	0	0	38	0
Lenalidomide	CLL/SLL	PMID: 28958469	1	III	1	1	317	1
	Systemic Light Chain Amyloidosis	PMID: 22504925	0	II	0	0	35	0
	Primary CNS lymphoma	PMID: 29986852	0	I	0	0	14	0
	Peripheral T cell lymphomas - Adult T cell leukemia/lymphoma (first - larger)	PMID: 23731832	1	II	0	0	54	0
	Hodgkin Lymphoma	PMID: 21937701	0	II	0	0	80	0
Sunitinib malate	Meningiomas	PMID: 25100872	0	II	0	0	50	0
	Thyroid carcinoma	PMID: 28301826	0	II	0	0	71	0
	Thymic Carcinoma	PMID: 25592632	0	II	0	0	57	0
	Chordoma	PMID: 19451429	0	II	0	0	53	0
Bendamustine hydrochloride	SCLC	PMID: 24736081	1	II	0	0	50	0
	Multiple Myeloma	PMID: 24270324	1	II	0	0	75	0
	Hodgkin Lymphoma	PMID: 27382096	0	II	0	0	59	0
	Peripheral T -cell Lymphoma	PMID: 23109692	0	II	0	0	45	0
	ALCL	*same trial as above						
	WM/LLT	PMID: 23433739	0	III	1	0	549	0
Everolimus	Glioma/Pilocytic	PMID: 23158522	1	III	1	1	117	0
	Meningiomas	PMID: 27311730	0	II	0	0	18	0
	Thyroid carcinoma	PMID: 24050953	0	II	0	0	40	0
	Endometrial Carcinoma	PMID: 25624430	0	II	0	0	31	0
	Gastrointestinal Stromal Tumor	PMID: 20507881	1	I/II	0	0	117	0
	Perivascular Epithelioid Cell Tumor	PMID: 22943457	N/A	N/A	N/A	N/A	1	N/A
	Thymoma	PMID: 29240542	0	II	0	0	41	0
	Thymic Carcinoma	*same trial as above						
	Osteosarcoma	PMID: 25498219	0	II	0	0	38	0
	Hodgkin Lymphoma	PMID: 20229590	0	II	0	0	19	0
	WM/LLT	PMID: 24716234	0	II	0	0	277	0
Pazopanib hydrochloride	Epithelial ovarian/Fallopian tube/Peritoneal cancer	PMID: 20584542	1	II	0	0	35	0
	Thyroid carcinoma	PMID: 20851682	0	II	0	0	152	0
	Uterine Sarcoma	PMID: 22595799	1	III	1	1	369	0
Eribulin mesylate	Uterine sarcoma	PMID: 26874885	1	III	1	0	452	1
Ipilimumab	SCLC	PMID: 27458307	1	III	1	1	1351	1
	Melanoma Brain Metastases	PMID: 29602646	0	II	1	0	76	0
	Uveal melanoma	PMID: 33417511	0	II	0	0	52	1
Vemurafenib	BRAF V600+ NSCLC	PMID: 26287849	1	II	0	0	208	0

	Brain Metastases	PMID: 27993793	1	II	0	0	146	0
	Thyroid carcinoma	PMID: 27460442	1	II	0	0	51	0
	Hairy Cell Leukemia	https://doi.org/10.1182/blood-2018-99-119949	0	II	0	0	30	0
Brentuximab vedotin	Diffuse Large B-cell lymphoma	PMID: 25573987	1	II	0	0	176	0
Pomalidomide	Primary CNS lymphoma	PMID: 30262659	0	I	0	0	29	0
	Systemic light chain amyloidosis	PMID: 22493299	0	II	0	0	378	0
Regorafenib	Osteosarcoma	PMID: 31013172	0	II	1	1	150	0
Cabozantinib s-malate	NSCLC	PMID: 27825636	0	II	0	0	86	0
Trametinib dimethyl sulfoxide + Dabrafenib mesylate	Rectal Cancer	DOI: 10.1200/jco.201.33.15_suppl.103	1	I/II	1	0	166	0
Trametinib dimethyl sulfoxide	Uveal melanoma	DOI: 10.1200/JCO.2016.34.15_suppl.9511	0	II	1	0	42	0
	Ovarian Cancer	PMID: 35123694	0	II/III	1	0	260	0
	Colon Cancer	DOI: 10.1200/jco.2015.33.15_suppl.103	1	I/II	1	0	166	0
Ibrutinib	Hairy Cell Leukemia	PMID: 33754642	0	II	0	0	44	0
	Primary CNS lymphoma	PMID: 28619981	0	I/II	0	0	109	0
Pembrolizumab	Brain Metastases	PMID: 27267608	0	II	0	0	65	0
	Prostate Cancer	PMID: 29992241	1	I	0	0	477	0
	Pancreatic Cancer	PMID: 28596308	0	II	0	0	113	0
	Occult Primary	PMID: 25891174	1	I	1	0	1260	0
	Anal Cancer	PMID: 28453692	1	I	0	0	477	0
	CLL/SLL	PMID: 28424162	0	II	0	0	65	0
	High-Risk Gestational Trophoblastic Neoplasia	PMID: 29185430	0	N/A	N/A	N/A	4	N/A
	Malignant Pleural Mesothelioma	PMID: 28291584	1	1	0	0	477	0
	Alveolar Soft Part Sarcoma	PMID: 29254498	0	N/A	N/A	N/A	50	N/A
	Undifferentiated Pleomorphic Sarcoma	PMID: 28988646	0	II	0	0	144	0
	Thymic Carcinoma	PMID: 29906252	0	II	0	0	33	0
	Bone cancer with mismatch repair	PMID: 28988646	0	II	0	0	144	0
	Mycosis Fungosis	PMID: 31532724	0	II	0	0	24	0
	Extranodal NK	PMID: 28188133	0	N/A	N/A	N/A	7	N/A
	Testicular Cancer	PMID: 29045540	0	II	0	0	12	0
	Uveal melanoma	PMID: 27533448	1	N/A	N/A	N/A	56	0
	Penile Cancer	PMID: 28596308	0	II	0	0	113	0
Nivolumab	Brain Metastases (melanoma, NSCLC Origin),	PMID: 29602646	0	II	1	0	76	0
	Richter Transformation	https://doi.org/10.1182/blood-2018-99-120355	1	II	0	0	74	0
	Merkel Cell Carcinoma	*No publication (NCT03071406)	0	II	1	0	50	0
	Extranodal NK	PMID: 28879531	N/A	N/A	N/A	N/A	3	N/A
	Uveal melanoma	PMID: 33417511	0	II	0	0	52	1
	Anal Cancer	PMID: 28223062	0	II	0	0	137	0
